# miR-204 mediated loss of Myeloid cell leukemia-1 results in pancreatic cancer cell death

**DOI:** 10.1186/1476-4598-12-105

**Published:** 2013-09-11

**Authors:** Zhiyu Chen, Veena Sangwan, Sulagna Banerjee, Tiffany Mackenzie, Vikas Dudeja, Xiaowu Li, Huaizhi Wang, Selwyn M Vickers, Ashok K Saluja

**Affiliations:** 1 Department of Surgery, University of Minnesota, Minneapolis, MN 55455, USA; 2 Department of Hepatobiliary Surgery, Southwest Hospital, Third Military Medical University, Chongqing, China

**Keywords:** Pancreatic cancer, miR 204, Mcl-1, Triptolide, Cell death

## Abstract

**Background:**

Pancreatic cancer is one of the most lethal human malignancies, with an all-stage 5-year survival of <5%, mainly due to lack of effective available therapies. Cancer cell survival is dependent upon up-regulation of the pro-survival response, mediated by anti-apoptotic proteins such as Mcl-1.

**Results:**

Here we show that over-expression of Mcl-1 in pancreatic patient tumor samples is linked to advancement of the disease. We have previously shown that triptolide, a diterpene triepoxide, is effective both *in vitro* and *in vivo*, in killing pancreatic cancer cells. Decrease of Mcl-1 levels, either by siRNA or by treatment with triptolide results in cell death. Using pancreatic cancer cell lines, we have shown that miR-204, a putative regulator of Mcl-1, is repressed in cancer cell lines compared to normal cells. Over-expression of miR-204, either by a miR-204 mimic, or by triptolide treatment results in a decrease in Mcl-1 levels, and a subsequent decrease in cell viability. Using luciferase reporter assays, we confirmed the ability of miR-204 to down-regulate Mcl-1 by directly binding to the Mcl-1 3’ UTR. Using human xenograft samples treated with Minnelide, a water soluble variant of triptolide, we have shown that miR-204 is up-regulated and Mcl-1 is down-regulated in treated vs. control tumors.

**Conclusion:**

Triptolide mediated miR-204 increase causes pancreatic cancer cell death via loss of Mcl-1.

## Background

Pancreatic cancer is the fourth leading cause of cancer related deaths in the United States with a five year survival of less than 5% [1]. Over 44,000 cases were diagnosed last year, and nearly the same number succumbed to the disease [2]. This dismal outcome is due to late stage diagnosis and lack of available chemotherapeutic options [3,4].

Cancer cells evade cell death by up-regulation of pro-survival pathways and down-regulation of cell death pathways. One of the protein groups involved in evasion of apoptotic cell death is the Bcl-2 superfamily [5,6]. Bcl-2 family members inhibit most types of apoptotic cell death, implying a common mechanism of lethality [7]. Mcl-1, a Bcl-2 superfamily member, has a critical role in regulating the balance between survival and death signals. It is over-expressed in human tumor tissue and promotes cell survival [8], and shRNA-mediated knockdown of Mcl-1 triggers apoptosis in lymphoma cells. Its importance in cell survival is underscored by studies associating over-expression of Mcl-1 with attenuated apoptosis induced by a variety of agents including quercetin, etopside, staurosporine and Actinomycin D [9-12].

Dysregulation of normal pathways allow cancer cells to thrive in a tumor promoting microenvironment. This loss of regulation can occur at the transcriptional, translational or post-translational levels. MicroRNAs typically act as tumor suppressors or oncogenes by binding to the UTR of their target gene and are involved in tumor formation and progression [13]. Mcl-1 is reported to be regulated by the miR-204 microRNA in head and neck squamous cell carcinoma (HNSCC), where it behaves as a tumor suppressor [14].

Recent research suggests that Mcl-1 not only regulates apoptotic cell death in response to certain chemotherapeutic agents, but is also responsible for inducing autophagy in some cells [15]. Although autophagy is a self-degradative process that is important for balancing sources of energy at critical times in development and in response to nutrient stress, some chemotherapeutic agents are capable of inducing cancer cell death through autophagy [16].

We and others have identified triptolide, a diterpene triepoxide derived from a Chinese plant, *Tripterygium wilfordii,* as a potential chemotherapeutic agent against pancreatic, breast and colon cancers, as well as cholangiocarcinoma, osteosarcoma and neuroblastoma [17-20]. Our group has shown that triptolide is capable of inducing apoptotic as well as autophagy as a mechanism of cell death in some pancreatic cancer cell lines [21]. Although triptolide is shown to be a very effective compound *in vitro*, its use in clinical settings is limited owing to its low solubility. We have therefore synthesized a water soluble pro-drug of triptolide, Minnelide, that has shown remarkable efficacy in pre-clinical studies. This compound prevents tumor formation and causes tumor regression of pancreatic tumors derived from cell lines of varying aggressiveness as well human tumor xenografts from patients [22]. However, the mechanism by which triptolide/Minnelide acts on pancreatic tumors is poorly understood.

In the current study we show that Mcl-1 over-expression correlates with advanced stage of disease. Down-regulation of Mcl-1 results in pancreatic cancer cell death, either via apoptosis or autophagy. Over-expression of miR-204, either by triptolide treatment or a miR-204 mimic transfection results in suppression of Mcl-1 expression and cell death, both in pancreatic cancer cells and human patient xenografts.

## Results

### Mcl-1 is over-expressed in human pancreatic cancer cell lines and tissue samples

**Figure 1:**
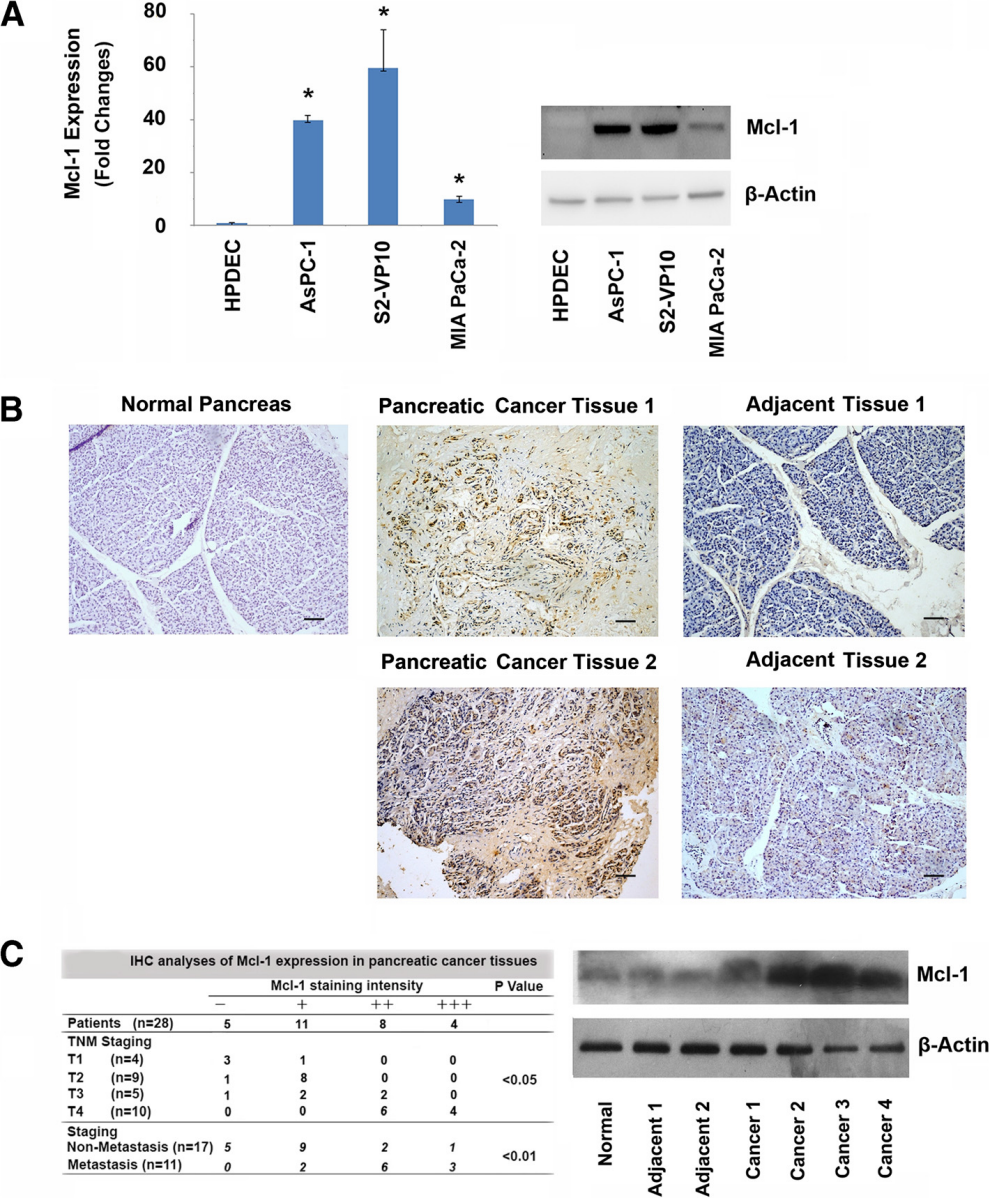
**Mcl-1 is over-expressed in pancreatic cancer cells and human pancreatic cancer tissues. A**. Pancreatic cancer cell lines show increased Mcl-1 levels compared with human pancreatic ductal epithelial cells at both the mRNA and protein levels. Left panel: RNA isolated from pancreatic cancer cells and normal ductal cells (HPDEC) show significantly increased expression of Mcl-1 (*, p < 0.01; n = 3) in pancreatic cancer cells compared with HPDEC cells. Expression of Mcl-1 was normalized against 18S. Right Panel: Immunoblot analysis of Mcl-1 expression in pancreatic cancer cell lines and human pancreatic ductal epithelial cells (HPDEC). β-Actin was used as a loading control. **B**. Immunohistochemical analysis of Mcl-1 expression in pancreatic cancer and its adjacent normal pancreatic tissue. (Top left: Normal Pancreas; Top middle: Patient 1 tumor T_2_N_0_M_0_; Top right: Patient 1 adjacent normal; Bottom middle: Patient 2 with liver metastasis T_2_N_1_M_1_; Bottom right: Patient 2 adjacent normal). Scale bar, 100 μm. **C**. Left, Mcl-1 expression was detected in 23 of 28 human pancreatic cancer tissues. All 11 cases of metastatic pancreatic cancer tissues, show Mcl-1 expression. In contrast, only 12 of 17 cases of non-metastatic pancreatic cancer tissues have Mcl-1 expression. The expression of Mcl-1 was correlated with pancreatic cancer metastasis (p < 0.05), TNM staging (p < 0.01), but not with tumor size, or differentiation. Right, human pancreatic cancer tissues show higher expression of Mcl-1 in pancreatic cancer tissues, compared with normal pancreas or adjacent normal pancreatic tissues from cancer patients. Protein lysates were immunoblotted for Mcl-1 and β-Actin used as a loading control.

Mcl-1 is an anti-apoptotic protein that is over-expressed in several cancers, but its expression in pancreatic cancer is poorly understood [23-25]. A previous report suggests that Mcl-1 is over-expressed in pancreatic adenocarcinoma [26]. We therefore assessed Mcl-1 expression in pancreatic cancer cell lines of varying aggressiveness (S2-VP10, AsPC-1, MIA PaCa-2) and compared the levels to that in normal human pancreatic ductal cells (HPDEC). Mcl-1 gene expression was higher in all cancer cell lines tested than HPDEC cells at both the RNA and protein levels (Figure 1A). Evaluation of Mcl-1 expression in human patient tumors show higher levels of Mcl-1 in tumor tissue compared to the adjacent normal tissue as well as normal pancreas (Figure 1B).

We further investigated the correlation between increased Mcl-1 expression and staging of the disease. Twenty-eight human pancreatic cancer sections were stained for Mcl-1 and expression was detected in 23 of 28 human pancreatic cancer tissues (82.14%). Further breakdown of these samples show that all of the cases of metastases were positive for Mcl-1 expression (11/11). In contrast, only 12 of 17 cases of non-metastatic pancreatic cancer tissues show Mcl-1 expression (Figure 1C, left). The expression of Mcl-1 correlated with pancreatic cancer metastasis (p < 0.05) and TNM staging (p < 0.01), but not with tumor size or differentiation status. Immunohistochemical data was supported by increased Mcl-1 protein expression in lysates from these samples compared to adjacent normal as well as normal pancreatic tissue (Figure 1C, right). These data, taken together, demonstrate that Mcl-1 is over-expressed in human pancreatic cancer cell lines and human patient tumors and its increased expression correlates with advanced disease.

### Mcl-1 knockdown decreases pancreatic cancer cell viability through apoptosis and autophagy

**Figure 2:**
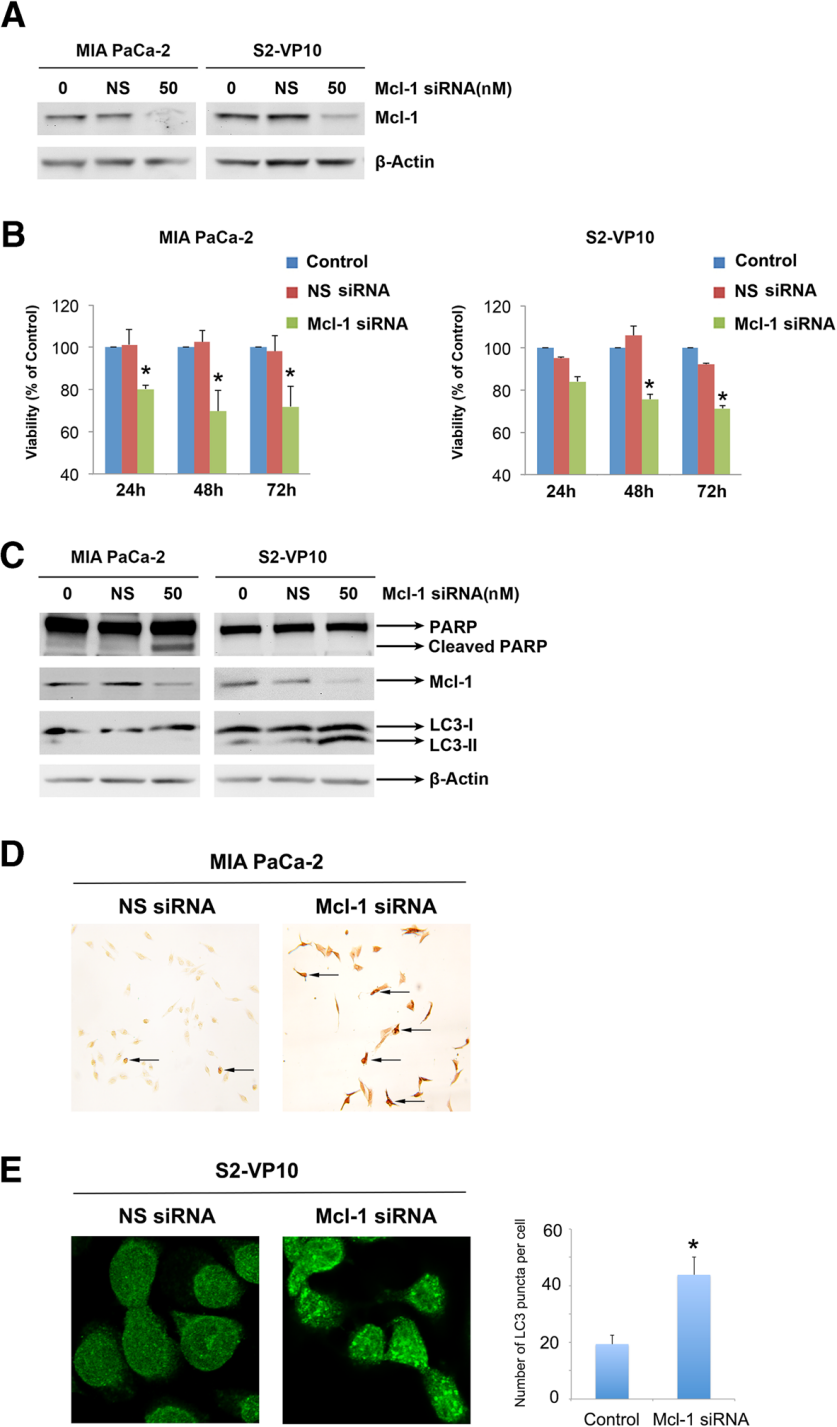
**Loss of Mcl-1 causes cell death in pancreatic cancer cells. A**. Mcl-1- specific siRNA causes loss of Mcl-1 protein in both MIA PaCa-2 and S2-VP10 cells. Protein lysates from Mcl-1 specific siRNA or non-silencing RNA were harvested and assessed for Mcl-1 knockdown. β-Actin was used as a loading control. **B**. Mcl-1 knockdown decreases viability in both MIA PaCa-2 and S2-VP10 cells. Cell viability was assessed by a WST-8 assay at times indicated and represented as% viability of untreated cells. Loss of Mcl-1 results in decreased cell viability in both cell lines. **C**. Lysates from Mcl-1 specific or control siRNA were assessed for levels of PARP, Mcl-1 and LC3-II 48 hours post-transfection. Mcl-1 siRNA treated MIA PaCa-2, but not S2-VP10 cells show PARP cleavage, a hallmark of apoptotic cell death. However, LC3-II, a marker for autophagy, was present in Mcl-1 siRNA treated S2-VP10 but not in MIA PaCa-2 cells. **D**. Mcl-1 siRNA transfection induces apoptosis in MIA PaCa-2 pancreatic cancer cells. Cell apoptosis was detected by TUNEL assay. Cells were transfected with Mcl-1 siRNA on day 2 and stained 48 hours after transfection. The arrow indicates apoptotic cells. **E**. Treatment of S2-VP10 cells with 50 nM Mcl-1 siRNA for 48 hours shows a significant increase in the LC3 punctate staining pattern when compared with untreated cells. Results shown are representative of 3 independent experiments.

To evaluate the role of Mcl-1 expression in survival of pancreatic cancer cells, we decreased levels of Mcl-1 using Mcl-1 specific siRNA. Cells were harvested 48 h after transfection, and efficacy of Mcl-1 silencing analyzed by Western blot (Figure 2A). Whereas Mcl-1-specific siRNA significantly down-regulated Mcl-1 expression, neither non-silencing siRNA nor mock-transfected cells had an effect on Mcl-1 expression. Inhibition of Mcl-1 using siRNA significantly decreased cell viability in both MIA PaCa-2 (% of mock: 71.92 ±9.75) and S2-VP10 (% of mock: 71.37 ± 1.15) cells after 72 h (Figure 2B). This loss in cell viability can occur through either apoptotic or non-apoptotic pathways.

We determined the induction of apoptosis by Poly-(ADP-ribose) polymerase (PARP) cleavage and terminal deoxynucleotidyl transferase-mediated dUTP nick end labeling (TUNEL) assay.

Autophagy was detected by monitoring the formation of microtubule-associated protein 1A/1B-light chain 3 (LC3). LC3 consists 2 forms: the cytosolic form LC3-I and the membrane-bound form LC3-II. When autophagy is induced, an increase of migrating band LC3-II can be seen by Western blotting. LC3 can also be detected by immunofluoresence; LC3-II stains with a punctate pattern whereas LC3-I has a diffused staining pattern. Forty eight hours after siRNA mediated Mcl-1 knockdown, PARP cleavage was observed in MIA PaCa-2 cells, but not in S2-VP10 cells indicating that apoptosis occurs in MIA PaCa-2 cells; however, LC3-II was present in S2-VP10 cells, but not in MIA PaCa-2 cells, indicating an onset of autophagy in these cells (Figure 2C). We used TUNEL to further confirm apoptotic cell death after Mcl-1 siRNA transfection. TUNEL-positive cells were quantitated. Mcl-1 siRNA transfection significantly promoted MIA PaCa-2 pancreatic cancer cells apoptosis (Figure 2D). We also use LC3 immunofluorescence assay to detect autophagy in S2-VP10 pancreatic cancer cells after Mcl-1 siRNA transfection. A homogenous cytosolic distribution of LC3 can be detected in untreated S2-VP10 cells (Figure 2E, left), which shifted to a punctate pattern after Mcl-1 siRNA transfection (Figure 2E, right).

We therefore conclude that siRNA-mediated Mcl-1 knockdown induces pancreatic cancer death through apoptosis in MIA PaCa-2 cells and autophagy in S2-VP10 cells.

## Mcl-1 is a target of miR-204 in pancreatic cancer cells

**Figure 3:**
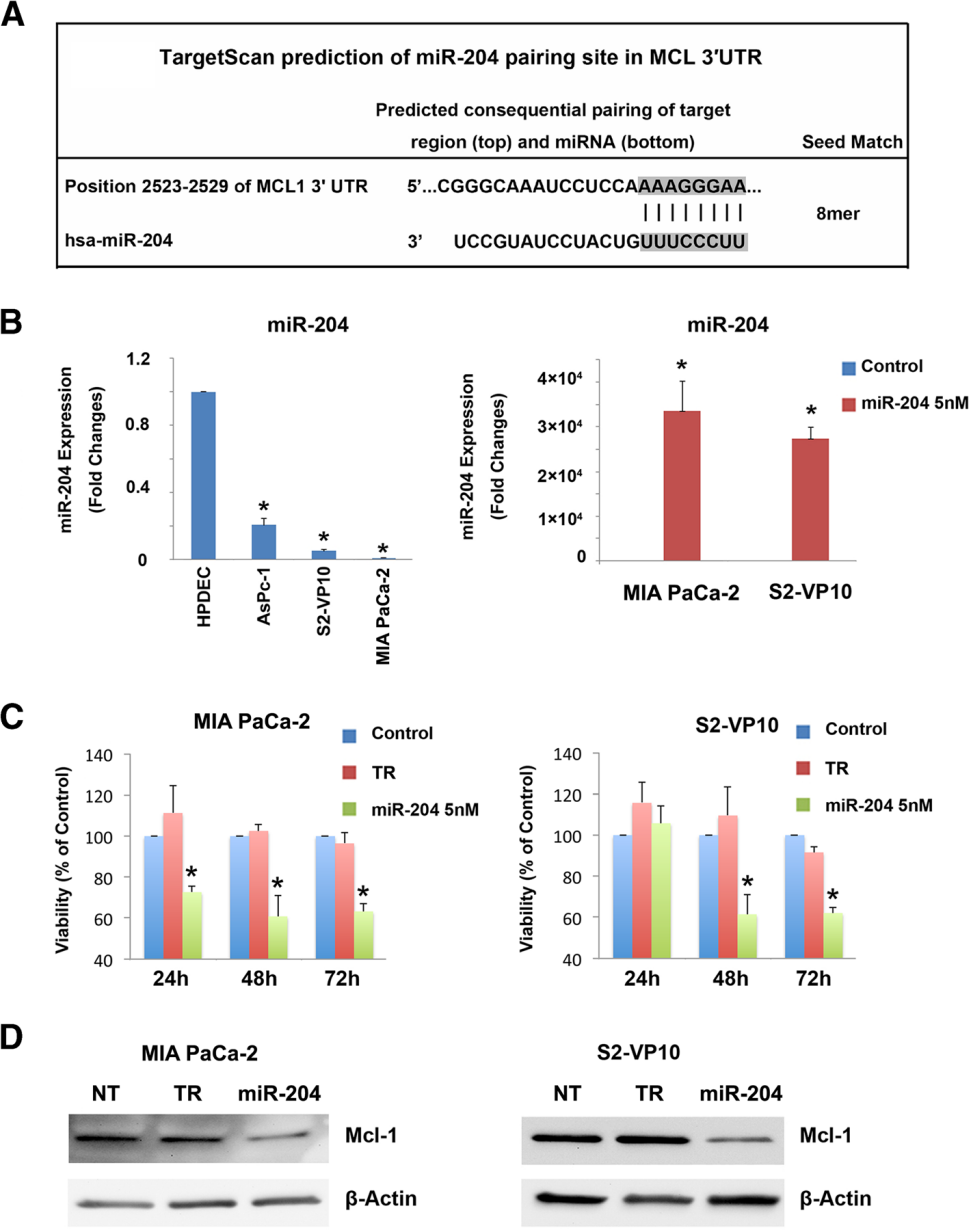
**Over-expression of miR-204 results in loss of Mcl-1 and causes cell death in pancreatic cancer cells. A**. Target Scan prediction of the miR-204 pairing site in Mcl-1 3’UTR. **B**. Left Panel. Pancreatic cancer cells show a decreased expression of miR-204, compared to HPDEC. Data represented as fold-change vs. HPDEC cells. miR-204 expression was measured by Real-Time PCR. Right Panel. Over-expression of miR-204 in MIA PaCa-2 and S2-VP10 cells transfected with a miR-204 mimic. **C**. Over-expression of miR-204 results in loss of cell viability in both MIA PaCa-2 and S2-VP10 cells. The bars represent mean ± standard error of the mean, n = 3. *p <0.05 (TR: transfection reagent only). **D**. Over-expression of a miR-204 mimic in MIA PaCa-2 and S2-VP10 cells results in reduced Mcl-1 protein expression 48 h post-transfection. β-Actin is used as a loading control (NT: non-transfected TR: transfection reagent only).

Once we had established that Mcl-1 is required for pancreatic cancer cell survival, we investigated the mechanism of regulation of Mcl-1. Using TargetScan 6.2, a database identifying putative miRNAs associated with mRNA, we identified Mcl-1 as a hypothetical target gene of miR-204 (Figure 3A). A previous study has shown that miR-204 is down-regulated in head and neck cancer [14], but there is no information available on the expression of miR-204 in pancreatic cancer cells. We therefore evaluated miR-204 expression using real time PCR in different pancreatic cancer cell lines (S2-VP10, AsPC-1, and MIA PaCa-2) and compared it to a normal pancreatic ductal cell line (HPDEC). Expression of miR-204 was lower in all cancer cell lines evaluated, compared to HPDEC (Figure 3B, left).

Since miR-204 was inhibited in pancreatic cancer cells, we assessed the effect of its up-regulation on cell survival. For this, we first over-expressed the miR-204 mimic in MIA PaCa-2 and S2-VP10 cells. Compared to control miRNA, miR-204 levels increased by 33493 ± 6754 and 27353 ± 2520 fold 48 h post-transfection in MIA PaCa-2 and S2-VP10 cells, respectively (Figure 3B, right). Once we had established that miR-204 levels were increased in the presence of mimic, we assessed cell viability in the presence of the mimic. Over-expression of miR-204 significantly decreased cell viability in MIA PaCa-2 and S2-VP10 cells 48 h after transfection (% of Control: 60.85 ±10.21 (MIA PaCa-2) and 61.48 ±9.48 (S2-VP10)) (Figure 3C).

Since microRNAs regulate gene expression leading to decreased translation, increased degradation of the target message, or both [27], we examined the effects of over-expression of miR-204 on Mcl-1 protein expression. In the presence of miR-204 mimic, Mcl-1 protein levels decreased, suggesting that miR-204 targets Mcl-1 in pancreatic cancer cells (Figure 3D). Our data therefore show that Mcl-1 over-expression in pancreatic cancer cells is due to down-regulation of miR-204.

### miR-204 binds to the Mcl-1 3’UTR

**Figure 4:**
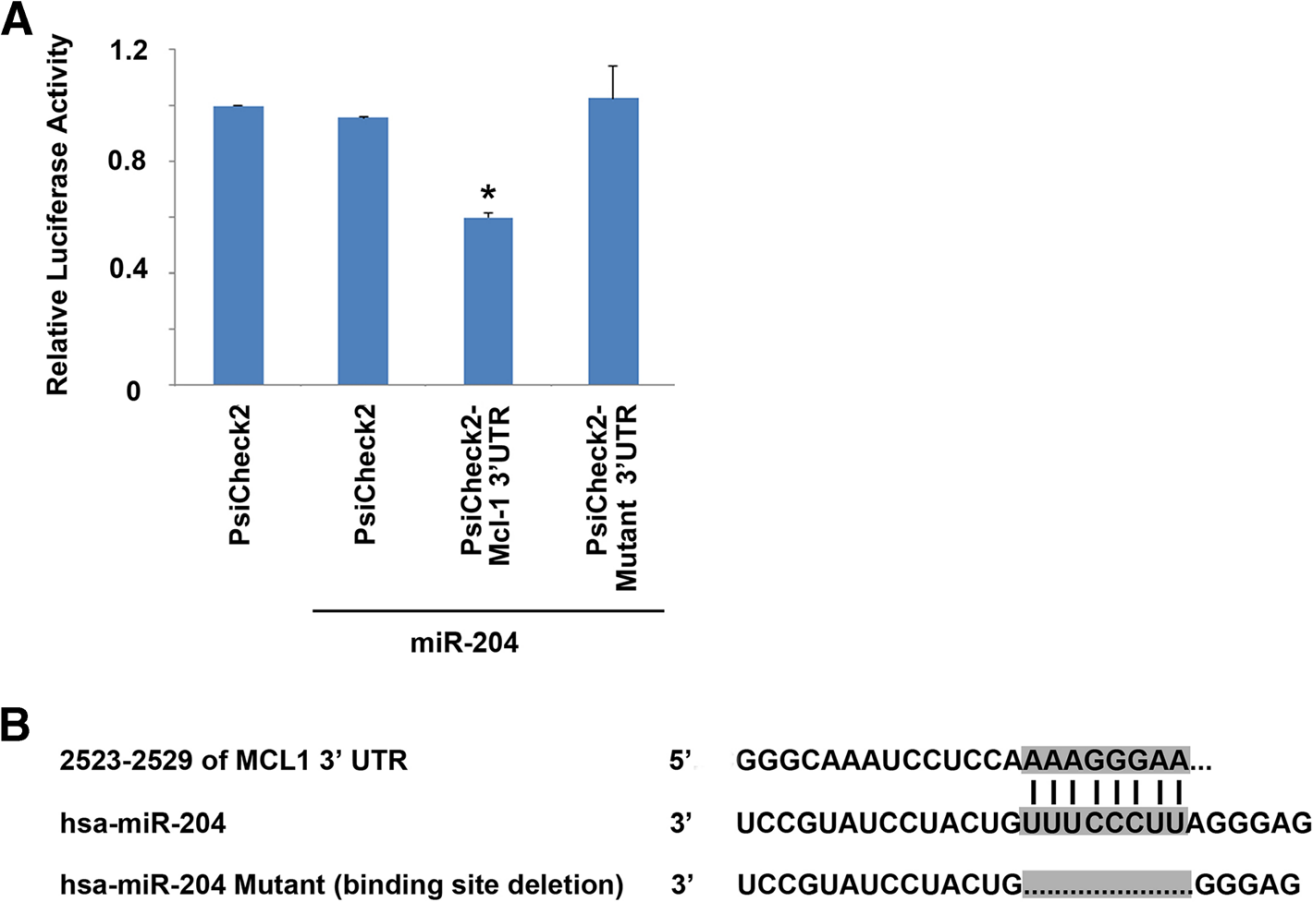
**miR-204 binds directly to the 3’ UTR of Mcl-1. A**. Luciferase activity in MIA PaCa-2 cells transiently transfected with the luciferase construct alone, or co-transfected with the vector containing either wild type or mutant miR-204 described in A. Mean ± SEM, *p < 0.01. **B**. Alignment of miR-204 with the predicted target region in the Mcl-1 3’UTR. The binding site deletion used to establish direct binding between miR-204 and Mcl-1 is shown. All constructs were cloned into the Psicheck2 vector.

Our data suggest that over-expression of miR-204 induces down-regulation of Mcl-1 in pancreatic cancer cells. To test if Mcl-1 expression was being regulated by miR-204, we transfected a fragment of the Mcl-1-3’UTR containing the miR-204 binding site in a Renilla/Luciferase reporter containing vector into MIA PaCa-2 cells in the presence of miR-204 or scrambled miRNA. Our data show that over-expression of wild type miR-204 abrogated reporter activity by 40% (Figure 4A), suggesting a direct interaction between Mcl-1 and miR-204. To validate binding specificity, we assessed reporter activity with a miR-204-Mcl-1 3’UTR binding site deletion mutant (Figure 4B). In the presence of the deletion mutant, no abrogation of reporter activity was observed, thereby confirming that miR-204 interacts directly with the 3’ UTR of Mcl-1 and inhibits the expression of Mcl-1.

### Triptolide regulates Mcl-1 and miR-204 expression in pancreatic cancer cells *in vitro*

**Figure 5:**
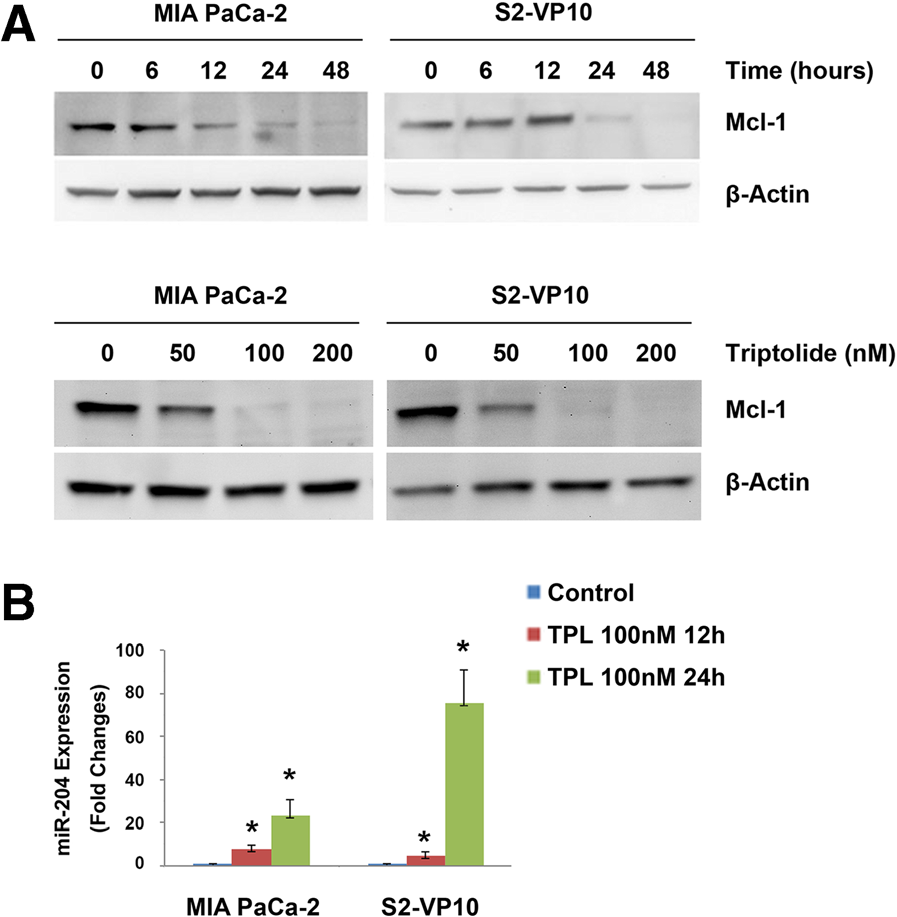
**Triptolide increases miR-204 and decreases Mcl-1 expression resulting in pancreatic cancer cell death. A**. Triptolide treatment of MIA PaCa-2 or S2-VP10 cells results in a time and dose dependent decrease of Mcl-1. β-Actin is used as a loading control. **B**. Treatment of both MIA PaCA-2 and S2-VP10 cells with triptolide significantly increases levels of miR-204 as assessed using real time PCR.

We have previously shown that triptolide, a diterpene triepoxide, is effective in causing pancreatic cancer cell death both *in vitro* and *in vivo*. Since Mcl-1 is up-regulated in pancreatic cancer and loss of Mcl-1 leads to cell death, we investigated whether triptolide decreases levels of Mcl-1 in these cells. Treatment of MIA PaCa-2 and S2-VP10 cells with triptolide showed a time and dose-dependent decrease of Mcl-1 protein (Figure 5A). In the presence of 50 nM triptolide, decrease in levels of Mcl-1 occurred between 6-12 h in MIA PaCa-2 cells but between 12-24 h in S2-VP10 cells. Correspondingly, triptolide treatment resulted in an increase in miR-204 levels in both MIA PaCa-2 (23.1 ± 7.5 fold) and S2-VP10 cells (75.2 ± 15.4 fold), 24 h post-triptolide treatment (Figure 5B). Treatment of cells with the same concentration of triptolide for 24 h did not lead to changes in miR-204 expression in normal ductal cells (HPDEC; data not shown). Taken together, our data show that triptolide treatment increased miR-204 levels and decreased Mcl-1 levels *in vitro*.

### Minnelide regulates Mcl-1 and miR-204 expression in pancreatic cancer cells *in vivo*

Minnelide, a water soluble pro-drug of triptolide, is shown to be extremely effective against pancreatic cancer both *in vitro* and *in vivo*[22].

**Figure 6:**
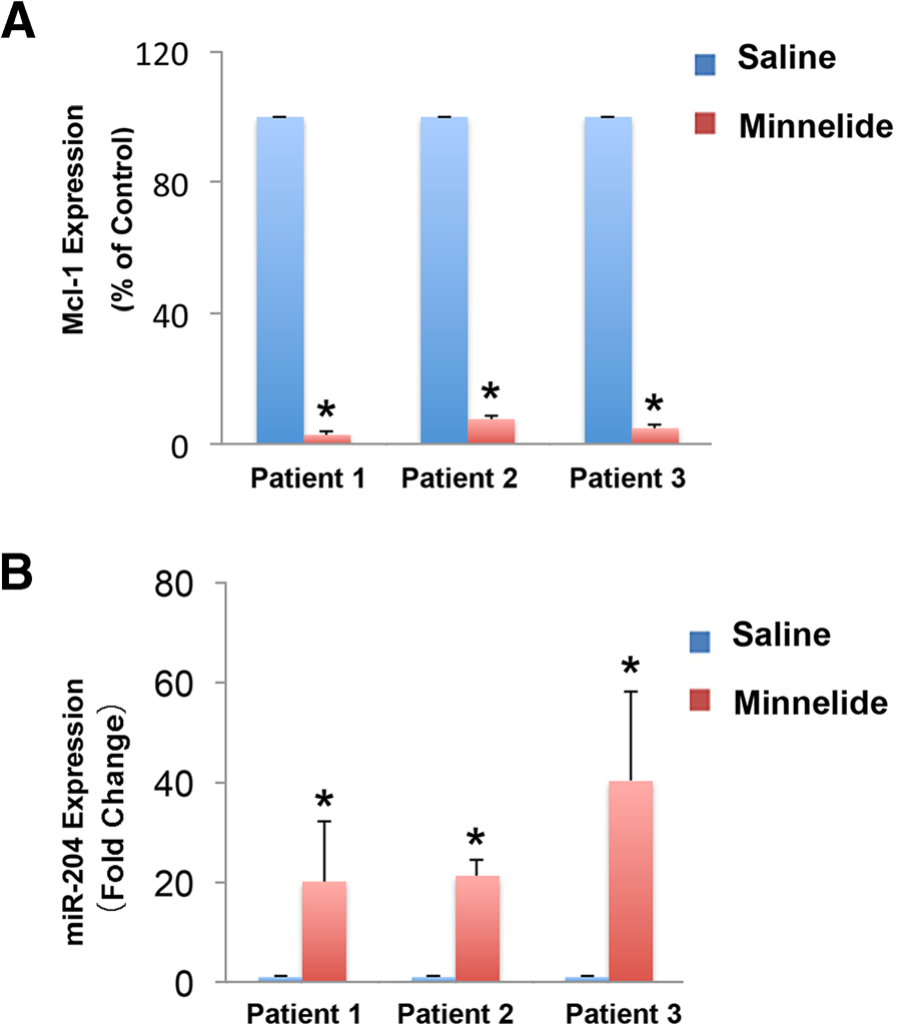
**Minnelide treatment leads to loss of Mcl-1 expression and increase of miR-204 *in vivo*. A**. Minnelide treatment decreases Mcl-1 levels in patient tumors transplanted into SCID mice. **B**. Minnelide treatment increases miR-204 levels in patient tumors transplanted into SCID mice.

To evaluate the ability of Minnelide to regulate Mcl-1 and miR-204 levels in a preclinical setting, we analyzed Mcl-1 and miR-204 expression in three patient tumor xenografts treated with Minnelide. Previous treatment with Minnelide for 40 days led to abrogation of tumors [22]. In order to study events occurring in response to Minnelide within a short time, animals bearing human xenografts were treated with Minnelide for seven days. Animals were then sacrificed and tumor samples collected. On day 7, there was a significant decrease (28.3% ± 3.8%) in tumor volume in all three patient tumors treated with Minnelide. mRNA from tumors treated with Minnelide had lower levels of Mcl-1 compared to saline treated tumors (2-7% of control tumors) (Figure 6A). In support of our *in vitro* data suggesting that triptolide leads to an increase in miR-204 levels and decreased Mcl-1 levels, miR-204 expression was significantly increased (20–40 fold) in Minnelide treated vs. control tumors (Figure 6B). Our data, taken together, suggests that triptolide induces pancreatic cancer cell death via down-regulation of Mcl-1 and increased expression of miR-204.

## Discussion

Resistance to conventional chemotherapy remains a significant obstacle in long-term survival of pancreatic cancer patients, and the mechanisms of recurrence and resistance remain poorly understood. Recent genome-wide research suggests that Mcl-1 is subject to increased gene copy number across more than two dozen cancer types [28]. Exploiting drug regimens targeting pathways that down-regulate Mcl-1 expression is therefore a current strategy in cancer therapy [29]. Increase in levels of Mcl-1 has been associated with advanced staging in breast, colon and lung cancers, but its status in pancreatic tumors remained poorly understood. Mcl-1 also protects cancer cells against cell death and is known to contribute to chemoresistance. Our results show Mcl-1 is up-regulated in pancreatic tumors but not in the adjacent normal tissue (Figure 1). Here we show that while Mcl-1 levels correlate with TNM staging and advanced stage of disease (Figure 1). Peddabonina et al. have recently shown that siRNA mediated loss of Mcl-1 results in decrease in cell viability in colon and lung cancers, and loss of chemoresistance [30]. In agreement with these studies, we show that loss of Mcl-1 by Mcl-1 specific siRNA results in cell death in both MIA PACA-2 and S2-VP10 pancreatic cancer cells (Figure 2).

MicroRNA based regulation of several pro-survival pathways have recently gained considerable interest. The function of miR-204, to date, is still unclear, although some mRNA targets that are important for normal cell development have been identified. miR-204 is reported to act as a tumor suppressor in a variety of cancers through different mechanisms including down-regulation of Bcl-2, NTRK2 in neuroblastoma cancer and suppression of invasion in endometrial cancer mediated by FOXC1 regulation [31,32].

Loss of miR-204 has recently been shown to promote cancer cell migration via increased expression of brain derived neurotrophic factor or its receptor, TrkB. Importantly, loss of miR-204 has been associated with a stem cell-like phenotype in gliomas, and its over-expression results in reduced tumorigenicity and loss of the stemness transcription factor, SOX4 [33]. Loss of miR-204 expression in gastric cancer has been associated with poor prognosis due to an increase in the anti-apoptotic protein, Bcl-2. In agreement with these studies, we have shown that miR-204 is down-regulated in pancreatic cancer cells, and over-expression of miR-204 induces loss of pancreatic cancer cell viability (Figure 3). While the role of miR-204 as a tumor suppressor is well established, its ability to regulate Mcl-1 expression was not known prior to this study.

Our previously published data has shown that triptolide-mediated cell death is cell-type dependent. While MIA PaCa-2 cells undergo apoptosis, S2-VP10 cells die via autophagy. Intriguingly, although the correlation between autophagy and tumorigenesis is well established, controversy about its pro-death or pro-survival role still exists [34]. In support of the role of autophagy as a cell death mechanism, caspase inhibition of L929 cells results in non-apoptotic, non-necrotic cell death. Additionally, knock down of Atg7 or Beclin-1 in these cells abrogates cell death. In the current study, we find that loss of Mcl-1 mimics triptolide mediated cell death; while MIA PaCa-2 cells undergo PARP cleavage, a hallmark of apoptosis, S2-VP10 cells show the presence of LC3-II, representing formation of autophagosomes (Figure 2). Previous studies have shown that high Mcl-1 level is an important factor for cancers to escape apoptosis [29]. However, little is known about Mcl-1 mediated protection against autophagy. A recent study has shown that cortical neuron-specific Mcl-1 deleted animals undergo autophagy, suggesting that Mcl-1 plays a role in both apoptosis and autophagy. However, the role of Mcl-1 in autophagic response of cancer cells is unclear. While there is some evidence to show that compounds that inhibit Mcl-1 expression cause autophagy-mediated cell death, no direct link between Mcl-1 and autophagic cell death has been shown until this study. VHL-regulated miR-204 is suppressed in VHL−/− renal clear cell carcinoma cells. Additionally, VHL expression increases miR-204 levels, resulting in down-regulation of LC3-II and cell death [35]. In our study, over-expression of miR-204 results in decrease in Mcl-1 expression and subsequent cell death in pancreatic cancer cells (Figure 3). Loss of Mcl-1 results in increased autophagy in S2-VP10 cells, but not in MIA PaCa-2 cells (Figure 2C). These data suggest that Mcl-1 regulation of autophagy may be cell line specific. Since the switch between pro-survival and pro-death autophagy is believed to be due to a shift in the balance of anti-apoptotic and pro-apoptotic protein expression, it would be interesting to evaluate the balance between the two in response to triptolide in S2-VP10 cells.

We and others have established that over-expression of Mcl-1 aids in cell survival and decrease in Mcl-1 levels results in cell death. We show in this study that one of the miRs that regulates Mcl-1 levels is miR-204. This is the first study demonstrating that triptolide increases miR-204 expression resulting in decreased levels of Mcl-1 by the direct binding of miR-204 to its 3’-UTR (Figure 4).

## Conclusion

In conclusion, in this study we provide a mechanism for triptolide induced cell death through regulation of miR-204. We have shown that triptolide up-regulates miR-204 and down-regulates Mcl-1, an anti-apoptotic protein essential for the survival of multiple cell lineages, and among one of the amplified genes in pancreatic cancer cells (Figure 5). This finding is also supported by the analysis of patient tumor xenografts treated with Minnelide, the water soluble prodrug of triptolide. Animals treated with doses of Minnelide shown to cause tumor regression show a decrease in levels of Mcl-1 and increase in miR-204 expression compared to saline treated controls (Figure 6). Therefore, an understanding of the mechanism of action of this prodrug will aid in establishing a treatment regimen for patient care in the near future.

## Materials and methods

### Cell culture

MIA PaCa-2 cells derived from a primary pancreatic tumor were obtained from ATCC and cultured in Dulbecco’s Modified Eagle Medium (DMEM) containing 10% fetal bovine serum and 1% penicillin-streptomycin. S2-VP10 cells (a gift from Dr. Masato Yamamoto, University of Minnesota) [36] were cultured in RPMI medium (Life Technologies, Carlsbad, CA) supplemented with 10% Fetal Bovine Serum and 1% penicillin-streptomycin. Ascites-derived AsPC-1 (ATCC) cells were cultured in Dulbecco’s modified Eagle medium containing 20% fetal bovine serum and 1% penicillin-streptomycin. All cells were maintained at 37°C in a humidified air atmosphere with 5% CO_2_. Human Pancreatic Ductal Epithelial Cells (HPDEC) (a gift from Dr. Craig D. Logsdon, MD Anderson, Texas) [37] were cultured in Keratinocyte Media (Life Technologies, Carlsbad, CA) supplemented with Bovine Pituitary Hormone (Life Technologies, Carlsbad, CA) and EGF (Life Technologies, Carlsbad, CA).

### Human samples

Twenty-eight pancreatic cancer patients from the hepatobiliary and pancreatic surgery department, Southwest Hospital, China were involved in this study. The tumor specimens included 11 metastatic pancreatic cancer (liver metastasis) specimens and 17 non-metastatic pancreatic cancers, as well as the appropriate adjacent normal tissue. Each pancreatic cancer specimen was reviewed by two pathologists. The research protocol was approved by the Institutional Review Board and all patients gave informed consent.

### Cell transfection

Syn-hsa-miR-204 miScript miRNA Mimic (MSY0000265) and FlexiTube human Mcl-1 short interfering RNA (siRNA) was purchased from Qiagen and used for transfection. Cells were seeded in 6-well or 96-well plates and incubated overnight prior to transfection. Mcl-1 siRNA or miR-204 mimic was transfected following manufacturer’s instructions. Cells were harvested 24 h post-transfection for mRNA analysis, and 48 or 72 h post-transfection for protein or cell viability assays.

### Immunohistochemistry

Deparaffinized tissue sections were trypsinized (0.05% trypsin with 0.05% Triton X-100) and blocked with 10% goat serum (Zymed Laboratories). Sections were incubated with the Mcl-1 antibody overnight at 4°C. The slides were then processed in the Ventana-automated stainer (Ventana Medical Systems) according to manufacturer’s instructions. Sections from normal pancreas were used as control. To correlate Mcl-1 expression with pathological parameters, the immunohistochemical findings were scored in a semi-quantitative fashion as previously described [38].

### Terminal deoxynucleotidyl transferase-mediated dUTP nick end labeling (TUNEL) assays

Forty-eight hours after 50 nM Mcl-1 siRNA transfection, cells were fixed with 4% paraformaldehyde (Sigma) solution in PBS for 1 h at room temperature, treated with 3% H_2_O_2_ in PBS, and then permeabilized with 0.1% Triton X-100 in PBS for 2 min on ice. The TUNEL assay (Roche Molecular Biochemicals) was carried out following the manufacturer’s instruction.

### Immunofluorescence

Cells were grown, treated with 50 nM Mcl-1 siRNA, and fixed as previously described [39], and stained using rabbit polyclonal anti-LC3 antibody (Cell Signaling) for LC3 staining. The LC3 dots were quantified using the Image J software command “analyze particles,” which counts and measures objects in thresholded images as we previously described [21].

### Determination of cell viability

Cell viability was determined by the WST-8 kit from Dojindo Labs. siRNA was transfected 18 h after cell seeding in a 96-well plate and viability assessed 24, 48 and 72 h after transfection. Briefly, 10ul of the tetrazolium substrate was added to each well and plates were incubated at 37°C for 1 h after which the absorbance at 450 nm measured. All experiments were done in triplicate and repeated at least three times.

### Quantitative real-time PCR

RNA isolation was performed using the mirVana RNA isolation kit (Ambion). cDNA synthesis was carried out using 1 μg of total RNA using the miScript II RT Kit (Qiagen) or High Capacity cDNA Reverse Transcription Kits (Applied Biosystems). Real-time PCR was performed using the miScript SYBR green PCR kit (Qiagen) according to the manufacturer’s instructions. Mcl-1 primers (F: TAAGGACAAAACGGGACTGG; R: CCTCTTGCCACTTGCTTTTC) were designed using the NCBI website. miR-204 (MS00003773, UUCCCUUUGUCAUCCUAUGCCU) primers were purchased from Qiagen. 18S and U6 were used as internal controls for quantifying Mcl-1 and miR-204 levels respectively (Qiagen). Relative levels of Mcl-1 or miR-204 were assessed using the ΔΔCt method [40].

### Dual-Luciferase reporter assay and 3’UTR binding site mutagenesis

MIA PaCa-2 and S2-VP10 cells (6 × 10^4^) were seeded in 24-well plates immediately prior to transfection. The Mcl-1-derived miR-204 binding site or a binding site deletion in the 3’UTR was inserted into the psiCheck2 expressing firefly luciferase plasmid (Promega) and transfected into MIA PaCa-2 or S2-VP10 cells using Attractene (Giagen) following manufacturer’s instructions. The miR-204 mimic was co-transfected where indicated. Forty-eight hours post-transfection, cells were assayed for both firefly and renilla luciferase using the dual luciferase glow assay (Promega).

### Human tumor xenograft model

Three de-identified human tumors were implanted subcutaneously into SCID animals (Jackson Laboratory). Once tumor size reached 500 mm^3^, tumors were dissected and cut into 10-mm^3^ pieces, which were then subcutaneously implanted into both flanks of additional SCID mice. One animal was treated with saline and the other with the water soluble prodrug of triptolide, Minnelide (0.42 mg/kg, QD) for 7 days. Animals were sacrificed 7 days after start of the treatment and RNA extracted from tumors was evaluated for Mcl-1 and miR-204 expression.

All experiments were performed in accordance with institutional guidelines and approved by the animal care and use committee at the University of Minnesota.

### Statistical analysis

All values are expressed as the mean ± standard error of the mean. All experiments using cell lines were repeated a minimum 3 times. Data for animal experiments represents tumors from three patients. Statistical significance was reported if p-value was < 0.05 using an unpaired Student t-test.

## Competing interests

The University of Minnesota has filed a patent for Minnelide, which has been licensed to Minneamrita Therapeutics LLC. Inventors on this patent include S.M.V., and A.K.S.. S.M.V. and A.K.S. have financial interests in Minneamrita Therapeutics LLC. The other authors declare that they have no competing interests. Minnelide synthesis has been filed under patent WO/2010/129918.

## Authors’ contributions

ZC and TNM performed the experiments and drafted the manuscript. XL and HW provided the human samples and supervised the immunohistochemical studies. VS, SB and VD participated in the design of the study and helped to draft the manuscript. AKS and SMV participated in the design and overall coordination of the study and helped to draft the manuscript. All authors read and approved the final manuscript.
